# Impact of *Yoga Nidra* on psychological general wellbeing in patients with menstrual irregularities: A randomized controlled trial

**DOI:** 10.4103/0973-6131.78176

**Published:** 2011

**Authors:** Khushbu Rani, SC Tiwari, Uma Singh, GG Agrawal, Archana Ghildiyal, Neena Srivastava

**Affiliations:** Department of Physiology, CSMMU UP, India; 1 Department of Geriatric Mental Health, CSMMU UP, India; 2 Department of Obstetrics & Gynecology, CSMMU UP, India; 3 Department of Statistics, Lucknow University, Lucknow, UP, India

**Keywords:** Menstrual disorders, psychological general wellbeing, *Yoga Nidra*

## Abstract

**Background:**

Yogic relaxation therapy (*Yoga Nidra*) has been effectively prescribed in conjunction with other medical and yogic procedures in the management of severe psychosomatic diseases, including cancer, bronchial asthma, colitis, peptic ulcer and menstrual irregularities.

**Aim of the study:**

To assess the impact of *Yoga Nidra* on psychological problems in patients with menstrual disorders.

**Materials and Methods:**

Patients were recruited from the Department of Obstetrics and Gynecology, C.S.M. Medical University (erstwhile KGMU), Lucknow, Uttar Pradesh, India. A total of 150 female subjects were randomly divided into two groups: 1) group of 75 subjects (with yogic intervention) and 2) a control group of 75 subjects (without yogic intervention). Assessment of psychological general wellbeing (tool) was used for all the subjects Assessment of psychological general well being (tool) was used for all the subjects (Cases and controls). This assessment was done twice first time in the beginning (baseline) and then after six months.

**Results:**

Anxiety decreased significantly (*P*<0.003) and depression decreased significantly (*P*<0.01) in the Yoga group. Positive wellbeing and general health improved significantly (*P*<0.02), and vitality improved significantly (*P*<0.01) after six months of Yoga therapy (*Yoga Nidra*) in the Yoga group compared with the control group.

**Conclusion:**

The current findings suggest that patients with menstrual irregularities having psychological problems improved significantly in the areas of their wellbeing, anxiety and depression by learning and applying a program based on Yogic intervention (*Yoga Nidra*).

## INTRODUCTION

Yogic (relaxation) techniques offer a means to reduce the physiological and psychological reactions to stress.[1,2] Current achievement-based, demanding and high-profile societies have incurred increased risks and vulnerability for stress-related chronic pain and other illnesses.[3] Yoga is one of the many popular techniques for achieving relaxation. Yoga has its origin in ancient India. Its original form consisted of spiritual, moral and physical practices.[4] The different relaxation techniques often lead to specific psychological and physiological changes termed as relaxation response.[5]

*Yoga Nidra* is one such effective technique, not only for physical or mental relaxation but also for preparing the mind for yogic discipline. *Yoga Nidra* is qualitatively different from relaxation. It is a ‘sleep’ where all the burdens are thrown off to attain a more blissful state of awareness, a relaxation much more intense than ordinary sleep. *Yoga Nidra* aims to focus the mind to achieve relaxation and increase wellness. Researches also indicate that *Yoga Nidra* can be used as a therapeutic technique to cure psychological disorders like anxiety, hostility, insomnia etc, and psychosomatic diseases like asthma, coronary heart disease, cancer, hypertension etc. *Yoga Nidra* is a successful therapy for both recent and longstanding psychological disturbances of all kinds, especially high anxiety levels and neurotic behavior patterns.[6]

Generally, women’s problems are neglected in our society, particularly so gynecological problems such as menstrual irregularities like excess or scanty flow etc. Menstrual dysfunction is a common problem in the population of the reproductive age group. Incarcerated women have high rates of amenorrhea (9%)and menstrual irregularity (33%); psychological factors, stress and deprivation were found to be associated with menstrual irregularities.[7]

There are several types of menstrual cycle problems related to irregular periods that affect the frequency of menstruation like Polymenorrhoea (frequent periods – with intervals of 21 days or less), Oligomenorrhoea (infrequent menstrual periods – interval between menstruation exceeds 35 days), Amenorrhea (absence of periods for more than 60 days in a woman of reproductive age: physiologically occurs during pregnancy and breastfeeding).

There are some other conditions which are related to pain during menstruation such as Dysmenorrhea (cramps or painful menstruation, involves menstrual periods that are accompanied by either sharp, intermittent pain or dull, aching pain, usually in the pelvis or lower abdomen), and irregular periods that affect quantity of menstruation, which are Hypomenorrhoea (a diminution of the flow or a shortening of the duration of menstruation) and Menorrhagia (an abnormally heavy and/- or prolonged menstrual period). Many females also complain of pain, anxiety, depression, fatigue, and vomiting during the menstrual cycle throughout their reproductive life.

In a study, authors studied the association of menstrual symptoms with anxiety and depression in a sample of 82 first-year female medical students. Nearly, half the subjects reported the frequent occurrence of at least one menstrual symptom that appeared to cause discomfort but did not interfere with performance.[8] *Yoga Nidra* has been reported to relieve pain associated with dysmenorrhoea and excessive levels of premenstrual tension.[9] However, no studies are available to as see the effect of *Yoga Nidra* on the psychological symptoms associated with menstrual irregularities.

The present study was planned to assess the effect of *Yoga Nidra* on psychological general wellbeing in patients with menstrual irregularities

## MATERIALS AND METHODS

### Study design and setting

We conducted a randomized controlled trial for women (menstrual irregularities) with psychological problems. Subjects were randomly assigned to a standardized six-month protocol of *Yoga Nidra* classes. We used computer-generated numbers for permuted block randomization. Treatment assignments were placed in opaque, sequentially numbered envelopes prepared by a biostatistician who had no contact with participants. The study was approved by the ethical committee at CSMMU U.P (erstwhile KGMU) Lucknow.

### Study participants

A total of 150 female subjects suffering from menstrual irregularities were included. Subjects with menstrual irregularities were diagnosed by a senior consultant in the Department of Obstetrics and Gynecology, Chatrapati shahuji Maharaj Medical University Lucknow, Uttar Pradesh, India. Participants were aged between 18 to 45 years with current menstrual irregularities persisting for more than six months. Interested individuals were initially screened for eligibility by a senior consultant after oral informed consent was obtained. According to the inclusion and exclusion criteria, eligible subjects were invited for yoga classes. At the first meeting, their socioeconomic history and menstrual history were recorded.

### Inclusion criteria

Women older than 18 years of age having menstrual irregularities, (pathological amenorrhea, dysmenorrhoea, oligomenorrhoea, polymenorrhoea, hypomenorrhoea, menorrhagia, and metrorrhagia) were included.

### Exclusion criteria

Women having known gynecological neoplasm requiring surgery, pregnancy, Pelvic Inflammatory Disease (PID) and uncooperative subjects were excluded.

### Data collection

The socio-demographic data, details of personal history and menstrual history (last menstrual period, duration of menstrual cycle, amount of bleeding and dysmenorrhoea) and pervious treatments such as physical therapy and yoga therapy were inquired and recorded. A total of 150 subjects were included and were randomly assigned to two groups: 1) Case Group of 75 subjects (with *Yoga Nidra* intervention and medication) and 2) Control Group of 75 subjects (only medication, without *Yoga Nidra* intervention). Medroxy progesterone, Norethistrone, Ethinyl estrodiol, Levonorgestrol, tranexemic acid, and Ethamsylate medicines were prescribed to the patients of both groups by the consultants as and where required. Out of 150 subjects, 10 subjects from Case Group and 14 subjects from Control Group were dropped because they did not turn up for the second follow-up. Out of 24 dropped patients, 16 patients could not follow the time schedule and eight patients did not feel better so were not willing after one week. Baseline assessments of all the subjects were carried out. Psychological General Wellbeing Index (PGWBI) was administrated to both the groups at the Commencement and after six months.

There were outcome measures:

1. Proforma for socio-demographic and personal history details.
2. Psychological General Wellbeing Index:[10] Translated Hindi version of PGWBI was used in the study. PGWBI consists of 22 self-administered items, rated on a 6-point scale, which assess the psychological and general wellbeing of respondents in six health-related quality of life (HRQoL) domains: anxiety, depressed mood, positive wellbeing, self-control, general health and vitality. Each domain is defined by a minimum of 3 or a maximum of 5 items. The scores for all domains can be summarized to provide a summary score, which reaches a maximum of 110 points, representing the best achievable “wellbeing”.

Procedure of translation of PGWBI:

- Three bilingual translators translated the original English version of PGWBI into Hindi independently.
- They discussed and compared the translated version item by item to agree upon a pre-final translated Hindi version (PFHV) of PGWBI.
- PFHV of PGWBI was tried on 10 literate and 10 illiterate women aged 18 years and above drawn from the community to observe the comprehensibility of the (PGWBI).
- Comprehensible version was taken as final translated Hindi version (FHV) of PGWBI.
- Two bilingual experts back-translated the FHV of PWBGI into English to establish meaning equivalence.
- The original English, the final Hindi version of PGWBI and the back-translated English version of the final Hindi version of PGWBI were referred to three other bilingual Mental Health professionals to assess the equivalence of the translated version of Instruments.
- These Mental Health professionals unanimously agreed that the three Instruments had very high-balancing meaning and lingual equivalence.

### *Yoga Nidra* intervention

We used the *Yoga Nidra* intervention, developed by Swami Satyananda Saraswati, School of Yoga, Munger, Bihar, India. The yoga instructor was selected by an expert panel for this study. The final protocol (yoga classes) consisted of 35 min per day, five days a week intervention in the department of physiology, CSMMU Lucknow, UP India for six months. Patients were subjected to *Yoga Nidra* intervention—a deep relaxation technique [Appendix 1].

### Data analysis

The differences in pre- and post-treatment scores were used for the analysis. This was done to take into account the imbalances, if any, at the baseline characteristics of the subjects. Student’s independent sample *t*-test (parametric test) was used to compare these differences in scores[12,13] between the two groups (yoga vs. non-yoga group) which were normally distributed. Statistical analysis was done using GraphPad inStat Version 3.05 software Inc year 2000.

## RESULTS

There were no significant differences for any of the socioeconomic or demographic variables, indicating that the random allocation was successful in ensuring equivalency in the socioeconomic demographic profile of the two groups [Table 1]. Likewise, no significant difference in baseline clinical characteristics between the two groups was found [Table 2].

**Table 1:** Socioeconomic demographic profile

	Mean±S.D	
	Cases (75) Yoga	Controls (75) Non yoga
Demographic profile of the subjects		
Age (Years)	28.53±7.07	27.62±7.78
Educational status of study subjects		
Illiterate	6 (8)	4 (5.33)
Undergraduate	24 (32)	27 (36)
Graduate	20 (26.67)	22 (29.33)
Occupational status of study subjects		
House wife	34 (45.33)	32 (42.67)
Desk worker	23 (30.67)	25 (33.33)
Field worker	18 (24)	18 (24)
Marital status		
Married	39 (52)	40 (53)
Unmarried	36 (48)	35 (46)

Figures in parentheses are in percentage

**Table 2:** Baseline characteristic of case and control groups

	N=75, Mean±S.D	
	Case	Control
Psychological variable		
Anxiety	13.92±3.83	14.17±4.3
Depression	9.46±2.59	9.54±2.7
Positive wellbeing	10.49±2.6	11.06±3.82
Self-control	9.98±2.57	10.13±2.69
General health	9.06±2.67	9.66±2.85
Vitality	11.14±3.7	11.86±3.49

### Principal outcome measures

Table 3 shows comparison of mean difference in score with Standard Deviation (SD) of pre-and post-treatment values Improvement in psychological symptoms. We found significant changes in anxiety (*P*<0.003), depression (*P*<0.01), positive wellbeing and general health (*P*<0.02) and vitality (*P*<0.01) in the case group after six months of yogic intervention with comparison to the control group. The *Yoga Nidra* was instrumental in causing improvement in self-control in experimental subjects in comparison to the control group but this was statistically not significant.

**Table 3:** Comparison of difference in pre- and posttreatment values of psychological variables in case and control groups

Variables	Difference in scores		t statistics	*P* value
	Case	Control			
	N=65 Mean±S.D	N=61 Mean±S.D			
Anxiety	1.38±0.84	0.96±0.70	3.00	0.003**
Depression	1.49.±0.86	1.08±0.91	2.57	0.01*
Positive wellbeing	1.33±0.92	098.±0.82	2.26	0.02*
Self-control	1.12±0.80	0.88±0.68	1.78	0.07***
General health	1.15±0.77	0.85±0.72	2.24	0.02*
Vitality	1.24±1.28	0.77±0.82	2.45	0.01*

** Very significant

* Significant

*** Not significant

### Adherence to the intervention

Out of 75 subjects in the case group, 65 subjects completed *Yoga Nidra* (at least 80% classes).

## DISCUSSION

The results suggest that the aspects of wellbeing studied improved in the case group. The participants in the *Yoga Nidra* program had decreased level of anxiety, depression and increased positive wellbeing, general health and vitality compared with the control group.

In the case group which practiced *Yoga Nidra* for six months, there was significant decrease in their degree of depression (according to the psychological general wellbeing schedule) and we observed a significant change during the treatment period. Other studies have also shown that those with depression could benefit from *Sudarshan Kriya* and related Practice.[14] Earlier studies also reported similar findings.[15,16] During depression, there is a decrease in neurotransmitters such as serotonin and norepinephrine. Besides, an increased level of cortisol has a role in causing depression by regulating the function of serotonin and norepinephrine.[17] Yoga helps in decreasing the cortisol levels leading to a counter-regulatory effect to reduce the depressive symptoms.[18]

The decrease in the anxiety level that a *Yoga Nidra* practitioner exhibits is interesting. There was significant decreased anxiety in the case group. Previous studies have also shown that employing yoga interventions for other conditions (cancer survivors, self-reported emotional distress) results in beneficial effects for depression and mood, as well as anxiety and physical wellbeing.[19] Many other studies.[20–23] have also supported our findings. There was a decrease in the anxiety level in the case group during the treatment period, whereas there were no changes in the control group. During anxiety there is an increased response of the hypothalamus and sympathetic activity. The *Yoga Nidra* state appears to reflect an integrated response by the hypothalamus, resulting in decreased sympathetic (excitation) nervous activity and increased parasympathetic (relaxation) function.[11]

The results show that there was a significant improvement in positive wellbeing, general health and vitality in the case group. *Yoga Nidra* is believed to balance psychic and vital energies within the psychic channels (nadis) of the energy framework underlying the physical body. Free flow of these energies is considered to be the basis of optimal physical and mental health.

Findings of other studies also corroborate the present results.[24–26] However, the improvement in self-control did not show a significant difference, but in the case group there was improvement in these parameters also when compared with the control group. Previous studies have found significant improvement in self-esteem[27] with Yogic exercises.

The subjects who practiced *Yoga Nidra* felt that they have learnt a skill in the form of *Yoga Nidra* that can be used in stressful situations to become relaxed and for better management of stress. They experienced that the yoga program helped in decreasing nervousness, tensions, depression, downheartedness, hopelessness, illness and bodily disorders. They felt happy, satisfied, cheerful and lighthearted. They experienced a new outlook of life. Furthermore, it could be that the energy was used to handle the feelings and emotions that they previously suppressed.

In this present study, psychological improvement in subjects with menstrual irregularities, secondary to implementation of *Yoga Nidra* appears to have the potential to generate positive effects on wellbeing.

The yogic exercise such as *Sudarshan Kriya* and related practices are known to increase experience of Altered States of Consciousness (ASC), similar to trans-state and this is manifested in a feeling of deep relaxation and release of stress.[28] *Yoga Nidra* has similar effects.

## CONCLUSION

The current findings suggest that patients with menstrual irregularities having psychological problems improved significantly in the areas of their wellbeing, anxiety and depression by learning and applying a program based on yogic intervention (*Yoga Nidra*).

## Appendix 1: Instructions *Yoga Nidra* were given in the following manner

- Preparation
  Get ready for *Yoga Nidra*. Lie down on your back on the floor and adopt the pose called shavasana. In this position the body should be straight from head to toe, the legs slightly apart and the arms a little away from the body, with the palms of the hands turned upwards. During *Yoga Nidra* there should be no physical movement (patient closes her eyes and keeps them strictly closed until is told to open) (pause). Take deep breath and as you breathe out feel the cares and worries of the day flow out of you. In the practice which follows, you are going to develop the feeling of relaxation in the body. During *Yoga Nidra*, you are functioning on the levels of hearing and awareness, and the only important thing is to follow the voice of the instructor (pause).
- Relaxation
  Now bring about a feeling of inner relaxation in the whole body; concentrate on the body and become aware of the importance of complete stillness (pause). Develop awareness of your body from the top of the head to the tips of the toes and mentally repeat the mantra O-o-o-m-m-m. Experience complete stillness and complete awareness of the whole body again O-oo- m-m-m (pause).
- Resolve
  At this moment you should resolve to yourself. The resolve will have to be very simple; it should be a short, positive statement in simple language stated three times with awareness, feeling and emphasis. The resolve you make during *Yoga Nidra* is bound to come true in your life (pause).
- Rotation of consciousness
  We now begin rotation of consciousness, rotation of awareness by taking a trip through the different parts of the body (pause), as quickly as possible the awareness is to go from part to part. Repeat the part in your mind and simultaneously become aware of that part of the body. Become aware of the right side, left side, back, front, and major parts of the body (pause).
- Breathing
  Become aware of your breath. Feel the flow of your breath in and out of your lungs (pause). Now concentrate your awareness on the movement of your navel area. Concentrate on your navel movements (pause). Your navel is rising and falling slightly with every breath, with each and every breath it expands and contracts (pause). Now start counting your breaths backwards from 27 to 1, like this: 27 navel rising, 27 navel falling, 26 navel rising, 26 navel falling, 25 navel rising, 25 navel falling, and so on. Say the words and numbers mentally to yourself as you count your breaths (pause).
- Image visualization
  Stop counting and leave your breath awareness; we now come to visualization. A number of different things will be named and you should try to develop a vision of them on all levels of feelings, awareness, emotions, imagination, as best you can.
- Resolve
  Now this is the time to repeat your resolve. Repeat the same resolve that you made at the beginning of the practice, do not change it, repeat the resolve three times with full awareness and feeling (pause).
- Finish
  Relax all efforts, draw your mind outside and become aware of your breath (pause), become aware of the natural breath, awareness of the whole body and awareness of breathing (pause). Your body is lying totally relaxed on the floor you are breathing quietly and slowly. Develop awareness of your body from the top of the head to the tip of the toes and say mentally in your mind O-o-o-m-m-m (pause). Please take your time, do not hurry. Start moving your body. When you are sure that you are wide awake, sit up slowly and open your eyes. The practice of *Yoga Nidra* is now complete.[11]
